# Comparative Performance of Rapid Diagnostics for the Detection of T-2 and HT-2 Toxins in Oats

**DOI:** 10.3390/molecules28186657

**Published:** 2023-09-16

**Authors:** Julie Meneely, Brett Greer, Oluwatobi Kolawole, Qiqi He, Christopher Elliott

**Affiliations:** 1 Institute for Global Food Security, National Measurement Laboratory: Centre of Excellence in Agriculture and Food Integrity, Queen’s University Belfast, 19 Chlorine Gardens, Belfast BT9 5DL, UK; brett.greer@qub.ac.uk (B.G.); oluwatobi.kolawole@qub.ac.uk (O.K.); q.he@qub.ac.uk (Q.H.); chris.elliott@qub.ac.uk (C.E.); 2 The International Joint Research Center on Food Security (IJC-FOODSEC), 113 Thailand Science Park, Pahonyothin Road, Khong Luang 12120, Thailand; 3 School of Food Science and Technology, Faculty of Science and Technology, Thammasat University, 99 Mhu 18, Pahonyothin Road, Khong Luang 12120, Thailand

**Keywords:** mycotoxin, T-2 toxin, HT-2 toxin, monitoring, rapid diagnostic kits, immunochemical methods

## Abstract

The contamination of oat crops by trichothecene mycotoxins, T-2 and HT-2 is an ongoing threat to our food safety. Within the industry, there are increasing concerns about the continued and growing presence of these mycotoxins occurring in oat crops due to climate change, farming practices and the handling of crops post-harvest. To safeguard human health, monitoring these mycotoxins in foodstuffs is paramount to ensure human exposure is limited. To achieve this, effective testing regimes must be established within the industry, consisting not only of rapid, reliable, and accurate analytical methods but also efficient sampling strategies. Four commercial rapid diagnostic kits were assessed against liquid chromatography coupled to mass spectrometry and included three lateral flow devices and one enzyme-linked immunosorbent assay. One-way ANOVA showed a *p*-value of 0.45 indicating no significant difference between the methods assessed. Qualitative analysis revealed test kits 1, 2, 3, and 4 showed false negative/false positive rates of 1.1/2.2, 7.6/0, 2.2/0, and 6.5/0 percent, respectively. Test Kit 1, the Neogen Reveal^®^ Q+ MAX for T-2/HT-2 Kit provided the most reliable, accurate and cost-effective results. Furthermore, its ease of use and no requirement for technical skill makes it applicable for on-site testing.

## 1. Introduction

Globally, the fungal contamination and the production of mycotoxins in staple crops such as wheat, barley, oats, and maize are a significant concern. They are of huge economic importance in terms of human and animal health as they cause serious adverse effects and in domestic and international agricultural trade [1,2,3]. Substantial economic losses arising from contaminated cereals include lower crops yields and product values, livestock losses due to animal health and productivity and the burden on human health [2]. In addition, costs relating to the management of mycotoxins such as regulatory compliance, preventions and mitigation strategies, and research are borne along the supply chain by producers, distributors, processors, and consumers [4].

Climate change will influence the growth of these mycotoxigenic fungi and mycotoxins and so they will continue to be important in terms of being one of the greatest threats to food safety [5,6]. Moreover, the geographical distribution of pathogenic fungi and consequently mycotoxin occurrence patterns will be altered [7]. While the global contamination of agricultural crops with mycotoxins above regulatory limits is approximately 25%, it has been shown that 60–80% of crops contain detectable levels [3].

Type A trichothecene mycotoxins are produced by a number of *Fusarium* species and include T-2 toxin, HT-2 toxin, T-2 Tetraol, T-2 Triol, Diacetoxyscirpenol, and Neosolaniol, although only T-2 and HT-2 are subject to regulatory control [8]. EU indicative limits for the sum of T-2 and HT-2 toxins of 1000 µg/kg and 200 µg/kg have been set for unprocessed and processed oats, respectively [8]; however, there are discussions underway to set maximum levels for the sum of T-2 and HT-2 in cereals and cereal products. These proposed maximum levels are 500 µg/kg and 50 µg/kg for unprocessed and processed oats, respectively [9,10], and will undoubtedly impact not only producers and processors but also the providers of rapid test kits. Although T-2 and HT-2 toxins have been detected globally in cereals such as maize, oats, wheat, barley, and rye, they are generally considered temperate climate mycotoxins [3]. A recent publication described the widespread occurrence of T-2 and HT-2 toxins in cereals in Spain, Italy, Latvia, Poland, Serbia, Romania, Hungary, Czechia, Croatia, Finland, and Sweden. Data from 2010 to 2019 indicated that the most heavily contaminated cereal with T-2 and HT-2 toxins was oats from Scandinavian countries, with contamination rates of greater than 60% in Finland and Sweden [11]. A study in Croatia from 2017 to 2018 revealed that contamination with T-2/HT-2 toxin was greatest in oats (70%), then barley (41%), maize (27%), and wheat (19%) [12]. High contamination rates of 95% and 98% were also reported for T-2 and HT-2 toxins, respectively, in milling oats in a European survey spanning harvests from 2013 to 2019 [10]. Furthermore, surveys of harvested oats in Ireland have indicated that T-2 and HT-2 toxins were the most frequently detected mycotoxins at incident rates of 41% and 51% [13] and 62% [14]. These studies underline the need for the regular testing of unprocessed cereals and cereal-based foods to ensure product safety and the quality and compliance with regulatory controls with respect to these toxins.

There are a wide variety of analytical tests available for mycotoxin testing along the supply chain. These range from sophisticated confirmatory/reference methods to rapid screening assays. The confirmatory methods generally use chromatographic separation (gas chromatography or high-performance liquid chromatography) coupled to mass spectrometry (GC-MS and LC-MS), ultraviolet (UV), flame-ionisation detection (FID), UV diode array (DAD), fluorescence, and electron capture [15]. These methods rely on considerable laboratory investment in terms of equipment and skilled personnel and are time-consuming and expensive to administer [16]. Therefore, for growers, suppliers, and processors along the supply chain, more user-friendly, inexpensive, and rapid techniques are favoured. However, these methods must be accurate, reproducible, and provide the required sensitivity for regulatory compliance.

To safeguard health by improving the quality of raw materials and their products, rapid tests have been increasingly promoted to validate food safety management systems used in the agri-food industry. In a survey conducted in 17 countries (11 in the EU and 6 non-EU), the authors reported that 66% of respondents used rapid test kits for an array of contaminants including mycotoxins [17]. This is further substantiated by the fact that, in 2020, the global mycotoxin testing market was estimated at USD 946 million and projected to reach USD 1337 million by 2025 [18]. Ultimately, this has been driven by several factors, namely legislative demands across many countries in the world often resulting in border rejections and product recalls, the increased contamination of products, climate change, and heightened awareness of consumers.

The rapid diagnostics market is hugely competitive and most of the tests available for T-2/HT-2 toxins are immunochemical methods including Enzyme Linked Immunosorbent Assays (ELISA), Lateral Flow Devices (LFDs)/Dipstick Assays and Fluorescence Polarization Immunoassays (FPIA). The companies providing these kits include Aokin AG (Berlin, Germany), Charm Sciences Incorporated (Lawrence, KS, USA), Elabscience Incorporated (Houston, TX, USA), Envirologix Incorporated (Portland, OR, USA), Eurofins Tecna Laboratories (Trieste, Italy), Hygiena LLC (Camarillo, CA, USA), Neogen Corporation (Lansing, MI, USA), Romer Labs Diagnostic GmbH (Getzersdorf, Austria), PerkinElmer Incorporated (Waltham, MA, USA), R-Biopharm AG (Darmstadt, Germany) and Vicam LP (Milford, CT, USA). That said, it should be noted that there are also multiplex immunoassay formats enabling the detection of several of the regulated mycotoxins. These include the Biochip Array Technology, (Randox Food Diagnostics, Crumlin, UK), for the measurement of nine mycotoxins [19], the flow cytometry instrument from Foss (Hillerød, Denmark) for the detection of six mycotoxins and the Myco 5-in-1 PLUS test produced by Vicam LP for the determination of up to six regulated mycotoxins.

The aim of this study was to select several commercially available rapid diagnostic kits (based on the claimed manufacturers’ performance) for the evaluation of their actual performance and therefore fitness for the purpose for analysis of the sum of T-2 and HT-2 mycotoxins in oats (unprocessed and processed). Three LFDs in addition to an ELISA, were selected for evaluation and comparison against state-of-the-art technology, LC-MS/MS. For growers, suppliers and processors along the oat supply chain, more user-friendly, inexpensive, and rapid techniques are favoured. However, these methods must be accurate, reproducible, and provide the required sensitivity for regulatory compliance. This study was commissioned by *safe*food with a view to helping the industry make informed choices in relation to what commercially available test kits are easy-to-use, applicable for on-site testing, and will provide accurate reliable screening results.

## 2. Results and Discussion

**Figure 1:**
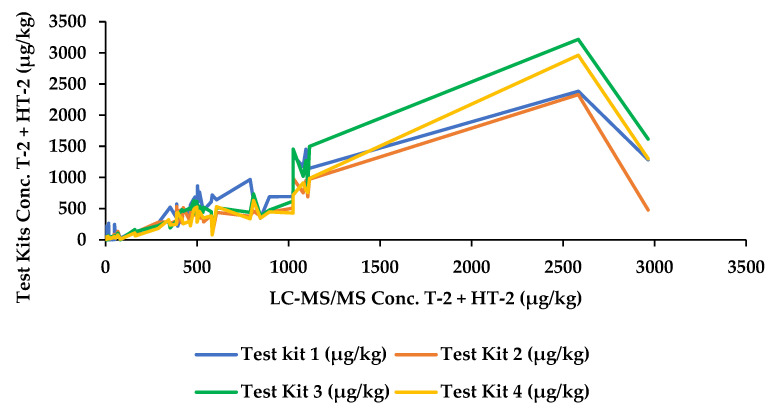
Comparison of the sum of T-2/HT-2 toxins by mass spectrometry and Test Kits 1–4.

In total, 100 oat samples (55 unprocessed, 44 processed, and 1 European certified reference material, T-2, and HT-2 toxin in oat flakes (ERM-BC270)) were analysed for the sum of T-2 and HT-2 using four commercially available rapid quantitative tests, in addition to confirmatory analysis by mass spectrometry. Concentrations (µg/kg) are detailed for all samples in Table 1 and a comparison of the mass spectrometry results with each of the commercial kits depicted in Figure 1. Eight samples were removed from the study owing to invalid results, i.e., the control line of the lateral flow device could not be seen, or if the resulting concentrations fell outside the kit range and no subsequent concentration value was obtained. Invalid results (i.e., the control line did not develop) were observed in Test Kit 1 and Test Kit 2 (samples 201 and 205, respectively). The number of samples showing results greater than the range was five, one measured using Test Kit 2 (sample 100) and four measured using Test Kit 3 (samples 71, 100, 134, and 157). According to the manufacturer’s performance characteristics, the quantification ranges for Test Kits 2 and 3 are 10–800 µg/kg and 50–500 µg/kg, respectively; therefore, although these samples were found to contain concentrations greater than the specified ranges, the results may still fall below the EU indicative limits for the sum of T-2 and HT-2 toxins so it could not be assumed that the samples were positive. Using Test Kit 4, two samples were determined to be less than the range (samples 167 and 198).

**Figure 2:**
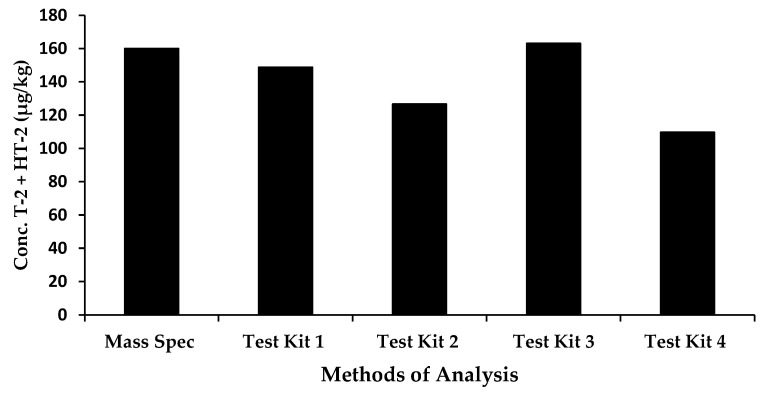
Comparison of rapid methods and mass spectrometry for the determination of the sum of T-2 and HT-2 toxin in a European Certified Reference Material for oat flakes.

ERM-BC270 was used as a quality control sample (single replicate) to also assess the performance of the kits. All oat samples were analysed using each kit in one run. The reference concentration value was 160 µg/kg for the sum of the T-2 toxin and HT-2 toxin. Figure 2 outlines the performance of the rapid methods compared with mass spectrometry.

The observed results of the certified reference material indicated that two rapid test kits, Test Kit 1 and Test Kit 3, performed very well, with concentrations of 149 µg/kg and 163 µg/kg obtained, respectively. Test Kits 2 and 4 displayed an under-estimation of the sum of T-2 and HT-2 toxins, (127 µg/kg and 110 µg/kg, respectively); however, this may be attributed to the cross-reactivity profile of the antibody included in the kits. For Test Kit 2, this information is unknown; however, for Test Kit 4, the antibody was raised against HT-2 toxin, therefore, the cross-reactivity with HT-2 toxin is 100% and that for T-2 toxin, 85%. This would account, in part, for the underestimation of the observed results of 110 µg/kg. Matrix effects may also contribute. It should also be noted that Test Kit 2 has not been validated in oats; therefore, some optimization would be required to improve the accuracy of results.

One-way ANOVA was used to determine whether the mean differences between the methods tested were significantly different. The *p* value of 0.45 indicated that the results obtained from each method were not significantly different, and therefore, provided no definitive answer as to which test(s) performed better.

Another method for examining the data would be to evaluate the qualitative results obtained for each method and calculate Cohen’s Kappa which measures the level of agreement between two sets of dichotomous scores. The samples were assigned as being negative or positive against the current EU indicative limits of 200 µg/kg and 1000 µg/kg for processed and unprocessed oats, respectively. The results for the comparisons of all commercial test kits against the confirmatory mass spectrometer results are displayed in Table 2. Additionally, included is Cohen’s Kappa for each. The interpretation of the result is as follows: K = <0 indicates no agreement or agreement equivalent to chance, K = 0.1–0.2 indicates none to slight agreement, K = 0.21–0.4 suggests fair agreement, K = 0.41–0.6 moderate agreement, K = 0.61–0.8 as substantial agreement, K = 0.81–0.99 as almost perfect agreement, while a value of K = 1 shows perfect agreement.

The results show that both Test Kits 1 and 3 displayed almost perfect agreement with LC-MS/MS; however, both Test Kits 2 and 4 displayed only fair agreement with K values calculated as 0.21 and 0.38, respectively.

### 2.1. Compliance/Non-Compliance in Relation to the EU Guidance Limits

The current indicative limits set by the European Union for processed and unprocessed oats are 200 µg/kg and 1000 µg/kg, respectively. To ascertain whether the performance of the kits met the current regulatory requirements, the results were evaluated in terms of being negative or positive against the current EU indicative limits, a qualitative approach. Table 3 shows the results for the methods assessed, compared with results from confirmatory mass spectrometry.

The results highlight that, generally, all kits tend to underestimate the concentration of the sum of T-2 and HT-2 in processed and unprocessed samples. For Test Kit 1 and Test Kit 3, the rates of false negatives/positives fall within the accepted tolerance of <5% [20]. However, for Test Kits 2 and 4, although the false positive rate is 0%, the false negative rate was determined as 7.6% and 6.5%, respectively. This increases the risk of contaminated cereals and cereal products entering the human food chain. However, it should be noted that, for Test Kit 2, three of the seven false negative sample results fell at ≥950 µg/kg, very close to the indicative limit. Depending on the cut-off limits determined, these may not be considered as negative samples and therefore the false negative rate of the kit would then be 4.3%, thus meeting the accepted criteria. For Test Kit 4, one sample out of the six false negatives found contained ≥950 µg/kg. If the same rationale were applied as for Test Kit 2, five false negatives would result; however, the 5.4% false negative rate would still fail the accepted tolerance. Given that Test Kits 2 and 3 have not been validated for the determination of T-2 and HT-2 in oats, the kits have performed very well. While Test Kit 3 met the false negative/false positive criteria, Test Kit 2 would require some optimisation to deliver increased accuracy.

The same qualitative evaluation was applied to the results in terms of the proposed new regulatory limits under discussion (i.e., 50 µg/kg for processed oats and 500 µg/kg for unprocessed oats). As observed from the summarised results (Table 4), the performance of the kits, in their current form, would fail to meet the criteria as laid down by the European Commission [20].

The only kit to meet the tolerance of ≤5% of false negatives was Test Kit 1; however, the rate of false positive results was 12%. In contrast, Test Kits 2 and 4 displayed low false positive rates of 4.3% and 2.2%, respectively, but did not meet the specifications for the rate of false negatives. Test Kit 3 failed to meet the criteria for the rate of false negatives, i.e., 7%, and displayed a false positive rate of 7.6%. Therefore, optimization and full validation would be required for all kits to meet the proposed regulatory limits. Furthermore, should the legislation change, the sensitivity of Test Kits 1 and 3 would need to be improved as the LOD for each is currently reported to be 50 µg/kg.

### 2.2. Test Kit Performance Characteristics

Comparing the methods has demonstrated other important aspects that are crucial for the cereal industry when monitoring both raw materials and finished products, i.e., the ease of use of the kit, time for analysis, and cost effectiveness. Test procedures for all LFD kits were extremely easy to follow and implement and could easily be conducted by non-trained personnel and may be performed in the field or on site. Sample preparation consisted of weighing out the sample, adding extraction powder and deionized or distilled water/buffer extraction solution and mixing. Following filtration and mixing with other buffers supplied, the sample extract is applied to the LFD. Sample preparation and testing takes minutes for completion. With respect to the ELISA kit tested, again sample preparation was similar to that for the LFD kits and was completed within minutes. For the test procedure itself though, many more steps are necessary, making it lengthier (45 min for test completion) and more complex to use. Furthermore, a level of expertise is required, as reliable results rely on the accurate pipetting of many reagents. Unlike portable LFDs, this test requires a laboratory situation. One advantage of the ELISA is that, because it is performed in a 96-well microtiter plate, the number of samples that can be analysed simultaneously is greater than that for LFDs.

The performance of the test kits is summarised in Table 5. Considering these characteristics, the recommended kit is Test Kit 1, the Neogen Reveal^®^ Q+ MAX for T-2/HT-2 Kit. Speed, cost per analysis, and the percentage rate of false negatives were amongst the most important features considered for on-site testing for the cereal industry. Test Kit 3 would be the second choice.

## 3. Materials and Methods

### 3.1. Chemicals and Materials

Analytical grade methanol was purchased from Sigma-Aldrich (Gillingham, UK). Ultra-pure water (18.2 MΩ-cm) was produced in-house using a Millipore water purification system (Millipore, Cork, Ireland). Test kits were purchased from Neogen Europe Limited (Ayr, UK), SGR Scientific Limited (Swords, Ireland), Bio-Check UK (St. Asaph, UK) and Fannin Ltd. (Dublin, Ireland).

### 3.2. Sample Collection and Preparation

Industry stakeholders provided samples for analysis, both unprocessed and processed oats. For unprocessed oats, samples for analysis were collected from static lots (lot size ≤ 1 ton), stored in warehouses and for processed grain (flake), samples were lifted directly from the conveyor feeding the packing machines in production. Then, 1 kg aggregate samples were supplied as detailed under current European Union legislation (Commission Regulation (EC) No 401/2006) [21]. For each lot, 10 incremental samples of 100 g were collected and combined to provide the aggregate sample. All samples (1 kg) were milled using a CGoldenwall multifunction grinder to a fine powder (particle size < 50 µm) and stored at −20 °C to preserve integrity, prior to analysis. The milled samples were homogenised in a paint shaker for 30 min prior to division by coning and quartering to achieve the test sample sizes required depending on the procedure to be used, (i.e., 10 g, 5 g, and 1 g). The number of samples selected and analysed was 100. One analyst performed the LC-MS/MS while another researcher tested the rapid diagnostic kits (blind study).

### 3.3. LC-MS/MS Analysis of Oat Samples

Sample analysis was performed using a fully validated method for the regulated mycotoxins: aflatoxins B_1_, B_2_, G_1_, G_2_, deoxynivalenol, ochratoxin A, zearalenone, T-2 toxin, HT-2 toxin, fumonisin B_1_ and fumonisin B_2_.

#### 3.3.1. Sample Extraction

A dilute-and-shoot sample extraction procedure was employed. Briefly, 1 g of finely milled material was weighed into a 15 mL centrifuge tube. Extraction solvent: acetonitrile: water: acetic acid (79:20:1, *v*/*v*/*v*) was added (5 mL) and the sample vortexed at 2500 rpm for 90 min using a multi-vortexer. Following centrifugation at 5000 rpm for 15 min, a 1 mL aliquot was removed and mixed 1:1 with acetonitrile: water: acetic acid (20:79:1, *v*/*v*/*v*) in an Eppendorf tube. The mixture was vortexed for 30 s and filtered through a 0.2 µm PTFE syringe filter into an amber LC-MS/MS vial for analysis.

#### 3.3.2. LC-MS/MS Parameters

Chromatographic separation was performed on an SCIEX ExionLC™ AD system with detection via SCIEX triple Quad 5500+ QTrap Ready LC-MS/MS system equipped with Turbo V™ ionisation source SCIEX, MA, USA). The mass spectrometer was operated in both positive and negative electrospray ionisation mode. Detection and quantification were accomplished using targeted analysis via a scheduled multiple reaction monitoring (sMRM). For each analyte, two MRM transitions were monitored, a precursor ion and two product ions. Details of these transitions and the operating conditions are outlined in Table 6.

Separation was achieved using a Gemini C18, 100 × 4.6 mm, 5 µm, 110 Å with the column maintained at 30 °C. Gradient elution used mobile phases of methanol/water/acetic acid 10:89:1 (*v*/*v*/*v*) (A) and methanol/water/acetic acid 97:2:1 (*v*/*v*/*v*) (B), both containing 5 mM ammonium acetate buffer were used. The binary gradient elution is detailed in Table 7.

A flow rate of 1.0 mL/minute was maintained with a sample injection volume of 5 µL. The total run time was 14.0 min.

Mass spectrometer parameters were as follows: curtain gas (CUR) = 35; collision gas (CAD) = 9; ion spray voltage: 4.5 kV (ESI+) and 4.5 kV (ESI−); temperature = 600 °C; ion source gas 1 (GS1) = 60 and ion source gas 2 (GS2) = 50.

#### 3.3.3. LC-MS/MS Validation and Method Performance

The validation of the method was based on publications by Sulyok et al. [22] and Steiner et al. [23]. An eight-point calibration curve was constructed for quantification. The concentration ranges were as follows: aflatoxins B_1_, B_2_, G_1,_ and G_2_, 0.025–12.5 ng/mL; deoxynivalenol, fumonisins B_1_ and B_2_, T-2 toxin, HT-2 toxin and zearalenone, 1–500 ng/mL; and ochratoxin A, 0.05–25 ng/mL.

The validation of the mycotoxin multi-method was performed using five different varieties of ‘blank’ oat samples in order to compensate for the lot-to-lot variation, similar to that described by Steiner et al. for complex feed [23]. The rationale for this protocol is that multimethod validations are commonly performed using a single lot of a matrix, as there are no particular regulations for this. However, not considering the intrasubject variation could give an additional component of uncertainty during the validation process. Therefore, this will be compensated for with the use of five different lots of oats (lot-to-lot variation) to add extra variability. These oat samples had initially been screened prior to validation in order to find oats samples negative for or which are below the LOD for the suite of mycotoxins in the method.

The validation of the method was performed following the SANTE/11312/2021 guidelines [24]. For method validation purposes, oat samples were spiked at two levels (Table 8) (with a factor of five difference) with the appropriate amount of the final working standard solution. Spiked oats samples were then left overnight at 4 °C to achieve solvent evaporation and to achieve equilibration between the matrix and analytes. The extraction of the spiked oats samples at both the high concentration level (HL) and low concentration level (LL), as well as the post-extraction spikes, were performed as outlined in Section 3.3.1, with 1 g of material extracted with 5 mL of extraction solvent. Each of the five oats samples were spiked in quintuplicate at the high concentration level (HL) on each day in order to assess the within laboratory reproducibility (WLR). The repeatability of the method was expressed as the relative standard deviation (RSD) calculated by spiking a set of five different oat samples in quintuplicate, and which is contrary to the “identical test items” as recommended in the CEN/TR 16059:2010 [25]. The determination of the matrix effects, expressed as the signal suppression/enhancement (SSE), and the recovery of the extraction step (RE), was performed by fortification of blank oats extracts at the high concentration level (HL) on day 3, all spiked in quintuplicate. Furthermore, in order to calculate the limit of quantification (LOQ) and limit of detection (LOD), each of the five different lots of oats samples were spiked in quintuplicate at the low concentration level (LL) on day 3.

Due to challenges in finding a matrix completely devoid of the suite of mycotoxins analysed, and therefore the inability to use matrix-matched calibration, validation was conducted through the use of a solvent (external) calibration curve. This is achieved through the serial dilution of the working standard solution with pure solvent. Furthermore, the definition of the term “recovery” in the guidelines mainly refers to the efficiency of the extraction process (RE) and does not consider the issue of matrix effects (SSE). This is particularly important when matrix-matching is not used, and therefore, the use of the term “apparent recovery” (RA) rather than the recommended definition in validation guidelines was used. This definition refers to both the matrix effects (SSE) and the efficiency of the extraction process (RE), and it is the “apparent recovery” that is used to calculate the repeatability (RSDr) and within-laboratory reproducibility (RSDWLR). In order to calculate the intra-day variation (RSDr), the concentrations calculated from the five different lots of oats spiked in quintuplicate on each day were averaged, equating to 15 replicates (*n* = 15) over the 3 days. For the within-laboratory reproducibility (RSDWLR), this was calculated based on measurements taken across all five different lots of oats spiked in quintuplicate across all 3 days, giving 75 replicates in total (*n* = 75). According to EC 2002/657 [26], the apparent recovery range applied in this work was 70–120%; however, the routine analysis permits recovery rates of between 60 and 140% [24]. Validation results are displayed in Table 9.

Method performance was also tested through participation in Proficiency Test Schemes through PTS accredited bodies. The performance of T-2 and HT-2 was evaluated through two such schemes, BIPEA: PT 31e: Mycotoxins—Feed; January 2023 and TRILOGY: Proficiency Test—TE-PT-MYC22-2—Mycotoxins; November 2022, yielding results’ z-scores of 1.91 and 0.1, respectively, for the sum of T-2 and HT-2.

### 3.4. Test Kit Analysis of Oat Samples

The kits used for this comparative study were commercially available rapid diagnostic kits and included three lateral flow kits and one ELISA kit.

**Figure 3:**
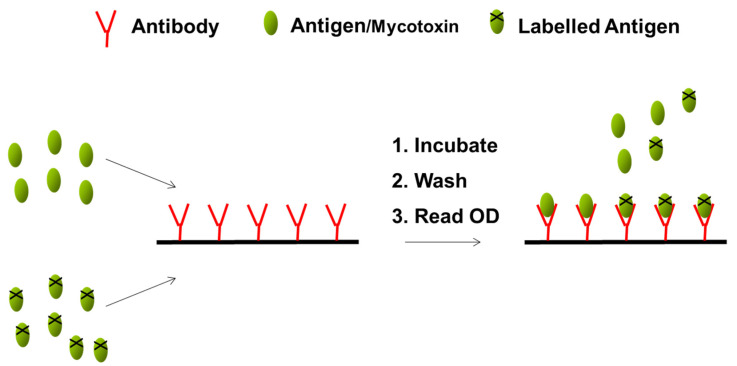
Schematic outlining the ELISA format.

Commercial ELISAs are commonly employed to screen cereals for mycotoxins. They allow the qualitative or quantitative measurement of mycotoxins in food and the principle is based on the use of specific antibodies and colour changes. Most often the assay is performed in microtiter plates. As mycotoxins are low molecular weight compounds, the assays are competitive. Figure 3 outlines the principle of the assay. Briefly, a pre-titrated concentration of the specific antibody is coated onto the wells of a 96-well microtiter plate. Sample or standard is added to the wells of the plate followed by the mycotoxin conjugated to an enzyme. Competition between unlabelled and labelled antigen (mycotoxin) for antibody binding sites occurs during a specified incubation period. After washing, to remove unbound material, the labelled bound antigen is measured by the addition of a suitable enzyme substrate producing a colour change. The absorbance reading is inversely proportional to the amount of toxin present [27].

**Figure 4:**
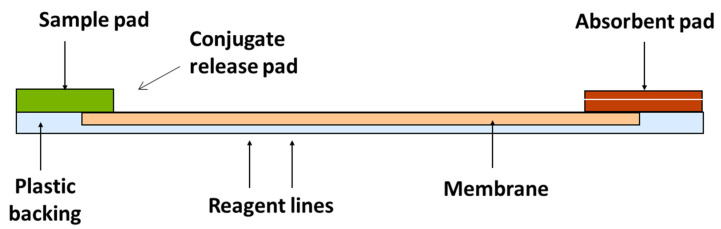
Components of an LFD.

LFDs also use the competitive format as described for ELISAs. They are composed of a number of different components outlined in Figure 4.

**Figure 5:**
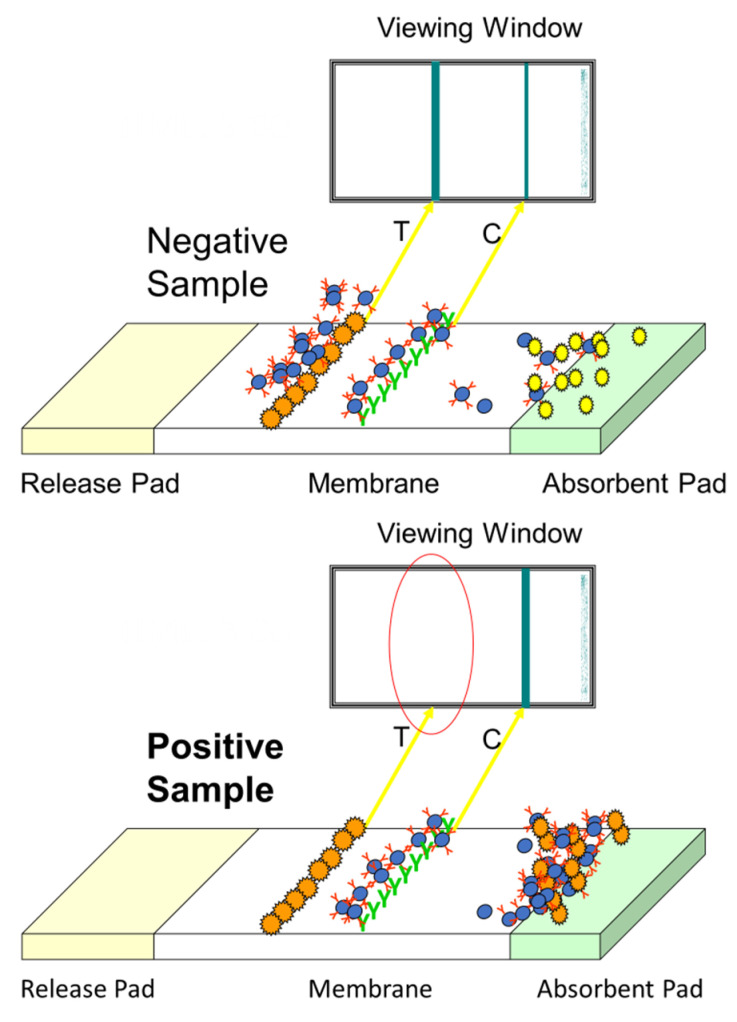
Schematic outlining the LFD format.

The extracted sample is applied to the sample pad and moves along the membrane by capillary action. Once it reaches the conjugate release pad, the dry conjugate (labelled antibody) is re-hydrated. If the analyte is present, it binds to the antibody and continues to flow along the strip. If no toxin is present, free antibody will bind to the test line (analyte of interest); therefore, the presence of a coloured line is inversely proportional to the amount of analyte present. The control line validates the test (composed of bound anti-species antibody) [27]. These rapid assays can either be qualitative, i.e., detecting the presence or absence of the toxin; or quantitative, providing a concentration level in the product [28]. The principle is shown in Figure 5.

The selection of test kits was based primarily on the ability to measure the sum of T-2 and HT-2, to meet the regulatory guidelines. LFDs were also the preferred option as they are rapid, easy to use, require few procedural steps, and may be used on-site. Preference was also given to those kits that used aqueous extractions, again facilitating their use on-site. Of the five commercially available LFD kits that met these criteria, three were selected. The two remaining kits were not available at the time of testing. Regarding the ELISAs, three of the commercially available kits were applicable to the quantification of T-2 and HT-2 toxin. One was selected as these are considered the ‘gold star’ in relation to screening assays.

**Figure 6:**
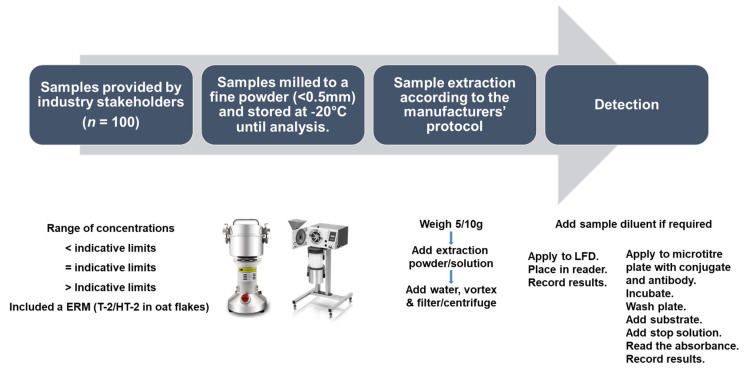
Schematic outlining the sample preparation, extraction, and analysis for the assessment of the commercially available rapid diagnostics.

Sample extractions and analyses were performed according to the kit manufacturers’ instructions. As sample preparation, extraction, and analysis were similar for all kit providers, a general scheme/protocol is outlined in Figure 6; however, the details of each protocol are also included in Table 10. The extraction powders/buffers provided by the manufacturers are proprietary information.

## 4. Conclusions

The aim of this study was to select several commercially available rapid diagnostic kits (based on claimed manufacturers’ performance) for an evaluation of their fitness for the purpose for analysis of the sum of T-2 and HT-2 mycotoxins in oats. Four commercially available rapid test kits were assessed against state-of-the-art LC-MS/MS. Although Test Kits 2 and 3 have not been validated for oats by the manufacturers, they were included in the study to assess their applicability to the oat matrix.

In terms of their performance, two of the commercially available LFD kits were shown to be fit-for-purpose for the quantification of the sum of T-2 and HT-2 in oats. The observed results indicated that, with respect to false negatives and false positives, Test Kits 1 and 3 fell within the accepted EU criteria (≤5%) for false negatives [20] and displayed low false positive rates. Moreover, the reliability of the test results (i.e., Test Kits 1 and 3 compared against mass spectrometry) were confirmed using Cohen’s Kappa. Values of 0.81 and 0.85, respectively, were calculated indicating excellent agreement with the confirmatory method. In contrast, Test Kits 2 and 4 did not meet the EU criterion for false negative rates, both exceeded 5%; however, false positive rates were 0%. Cohen’s Kappa values were determined to be 0.21 and 0.38, respectively, revealing much less agreement between the results produced by the screening tests when compared with LC-MS/MS. For on-site testing, LFDs are definitely the kits of choice. This evaluation has shown that reliable, accurate results that meet regulatory requirements may be obtained using these easy-to-use rapid methods and can therefore be implemented with ease by growers, suppliers, and processors along the supply chain to ensure compliance. For the implementation of these rapid immunodiagnostics, it is recommended that industry perform their own validation and establish a cut-off/threshold value in terms of compliant/suspect samples. Industry inter-laboratory comparisons would be beneficial to not only assess commercially available test kits for the analysis of T-2 and HT-2 but also to ensure that those performing the analyses are proficient in their use. This would help the industry in their decision-making processes regarding the monitoring of cereals for contaminants.

## Figures and Tables

**Table 1:** Results of processed and unprocessed oat samples using mass spectrometry and the four commercial rapid test kits.

Sample ID	Sample Type	LC-MS/MS	Test Kit 1	Test Kit 2	Test Kit 3	Test Kit 4
			(µg/kg)			
1	Processed	6.2	17.8	13.5	35.2	10.5
2	Processed	7.8	23.8	16.4	33.4	20.7
3	Processed	7.6	37.4	20.6	36.5	14.1
5	Processed	33.2	27.1	38.9	48.2	24.5
6	Processed	14.3	0	28.2	38.2	15.1
7	Processed	0.5	0	0	27.9	8.6
8	Processed	49.4	246.2	48.5	62.2	41.0
9	Processed	1.0	0	15.8	0	8.2
10	Processed	18.0	31.8	36.9	53.5	26.6
11	Processed	26.7	0	31.8	35.4	24.7
12	Processed	7.1	0	16.5	32.3	16.5
13	Processed	1.8	0	10.6	26.5	17.8
14	Processed	6.8	0	15.8	37.5	22.2
15	Processed	9.1	0	18	36.8	15.3
16	Processed	38.8	13.9	38.1	40.6	29.6
17	Processed	11.7	0	18.4	34.1	17.6
18	Processed	22.4	6.3	37.3	40.9	34.0
19	Processed	16.1	11.8	15.8	33.4	22.0
20	Processed	38.0	0	47.9	53	36.1
21	Processed	16.2	29.4	22.3	38.2	18.9
22	Processed	5.2	1.4	23.5	26.2	13.0
23	Processed	49.4	0.7	42.2	51.8	31.2
24	Processed	22.7	0	25.5	40.6	31.7
25	Processed	13.7	8.9	8.6	37.3	29.0
27	Processed	46.9	5.4	43	53	33.4
28	Processed	22.7	40.3	31.2	45.7	12.9
29	Processed	4.2	69.3	6.7	27	45.4
30	Processed	20.7	43	40.8	43.6	20.1
31	Processed	9.5	101.5	19.2	29.2	18.0
32	Processed	3.3	216.7	9.7	27.4	14.5
33	Processed	0.0	0	10.9	0	13.5
34	Processed	2.9	4.7	15.3	27.8	44.0
35	Processed	16.3	62.9	12.7	29.2	12.6
36	Processed	1.0	0.92	5.3	0	23.4
37	Processed	15.3	8.5	24.1	33	15.2
38	Processed	2.0	0	15.3	0	8.0
39	Processed	4.7	0.47	0	0	8.4
40	Processed	0.0	0	9.8	0	10.6
41	Processed	1.2	0.89	8.5	0	6.8
42	Processed	3.8	0	14.4	28.6	16.4
43	Processed	0.2	3.5	0	0	8.5
45	Processed	42.2	42.5	40.2	43.2	29.5
48	Processed	3.0	0	1.2	0	18.0
50	Processed	0.0	80.9	0	0	6.9
53	Unprocessed	790.4	966	377.2	439.4	336.4
56	Unprocessed	1105.1	1166.5	690	864	758.2
57	Unprocessed	404.2	319.7	471.8	429.6	380.9
58	Unprocessed	895.3	690.5	442.4	476.2	448.0
59	Unprocessed	353.2	523	261.4	190	237.1
63	Unprocessed	488.8	686.5	395.2	420.2	509.4
68	Unprocessed	513.7	764.5	351.4	540.2	452.1
71	Unprocessed	839.6	1122.5	966.1	>range	641.8
72	Unprocessed	45.8	76.8	84.6	55.4	49.9
74	Unprocessed	506.7	608	424.3	542.6	381.1
76	Unprocessed	13.7	0	7.2	0	16.9
77	Unprocessed	578.0	610	394.3	449.8	383.4
80	Unprocessed	582.9	717	80.5	94	78.4
81	Unprocessed	482.3	656.5	561.5	628.6	484.7
83	Unprocessed	1024.8	694	506.8	619.5	429.7
85	Unprocessed	388.8	572.5	532.6	346.1	425.4
86	Unprocessed	387.3	355	303.8	307.1	247.8
88	Unprocessed	536.2	474.5	286.2	521	350.9
90	Unprocessed	1025.0	1354.5	986	1454.4	718.7
93	Unprocessed	395.0	217.5	283.5	368	437.7
96	Unprocessed	1080.0	1173.5	755.1	1020	913.0
100	Unprocessed	0.0	1129.3	>range	>range	5092.4
101	Unprocessed	55.4	21.1	42.1	37.1	28.4
108	Unprocessed	80.9	21.8	0	0	10.2
116	Unprocessed	8.2	1.2	0	0	28.6
117	Unprocessed	458.2	416	317.9	503.8	297.5
118	Unprocessed	417.8	487.5	270.3	481.4	337.7
130	Unprocessed	0.0	0	6.1	0	19.0
134	Unprocessed	3993.3	1477	1461.5	>range	2919.4
137	Unprocessed	503.1	863.5	360.5	496.4	302.2
142	Unprocessed	463.6	560.1	425.1	490	223.9
148	Unprocessed	162.7	111.7	116.6	129	62.4
150	Unprocessed	14.3	124.9	33.6	65.2	54.5
151	Unprocessed	66.8	134.8	133.1	105.8	75.0
154	Unprocessed	1114.6	1148	972.7	1497.9	985.5
155	Unprocessed	808.5	625.5	461.3	736.2	629.5
157	Unprocessed	768.0	1319	676.8	>range	694.8
164	Unprocessed	424.7	478	515.9	457.6	256.1
165	Unprocessed	9.4	36.1	46.3	50.2	28.5
167	Unprocessed	1427.7	487	62.1	172.8	<range
169	Unprocessed	288.4	268.6	281.4	227.3	181.3
178	Unprocessed	532.2	490	348.9	438.6	359.0
179	Unprocessed	1095.1	1452	949.9	1271.4	796.6
180	Unprocessed	499.8	369	313	296.6	282.0
184	Unprocessed	846.3	344.5	378.6	383.8	346.6
189	Unprocessed	606.6	642	440	514.8	530.9
195	Unprocessed	2583.0	2384.5	2331.1	3218	2962.2
198	Unprocessed	1458.6	562.5	12.8	132	<range
201	Unprocessed	553.2	Invalid	396.9	517	317.0
205	Unprocessed	1383.5	832	Invalid	1011.2	905.9
216	Unprocessed	499.3	490.7	515	614.8	525.8
218	Unprocessed	345.0	489.6	288.4	303.9	321.0
223	Unprocessed	0.0	0	8	0	8.1
227	Unprocessed	2964.6	1284.5	477.2	1616	1299.4
229	Unprocessed	17.5	264.1	42.1	42.5	30.6
ERM	Processed	160	148.8	126.6	163.1	109.7

**Table 2:** Agreement of commercial Test Kits 1–4 with LC/MS-MS.

		LC-MS/MS		
		Positive	Negative	Total
Test Kit 1	Positive	7	2	9
	Negative	1	82	83
	Total	8	84	92
Cohen’s Kappa (K)		0.81		
Test Kit 2	Positive	1	0	1
	Negative	7	84	91
	Total	8	84	92
Cohen’s Kappa (K)		0.21		
Test Kit 3	Positive	6	0	6
	Negative	2	84	86
	Total	8	84	92
Cohen’s Kappa (K)		0.85		
Test Kit 4	Positive	2	0	2
	Negative	6	84	90
	Total	8	84	92
Cohen’s Kappa (K)		0.38		

**Table 3:** False positive/false negative rates for the rapid test kits (existing indicative limits).

Rapid Test Kit	Number of False Positives	False Positive Rate (%)	Number of FalseNegatives	False Negative Rate (%)
Test Kit 1	2	2.2	1	1.1
Test Kit 2	0	0	7	7.6
Test Kit 3	0	0	2	2.2
Test Kit 4	0	0	6	6.5

**Table 4:** False positive/false negative rates for the rapid test kits (proposed regulatory limits).

Rapid Test Kit	Number of False Positives	False Positive Rate (%)	Number of False Negatives	False Negative Rate (%)
Test Kit 1	11	12	3	3.3
Test Kit 2	4	4.3	13	14.1
Test Kit 3	7	7.6	7	7.6
Test Kit 4	2	2.2	11	12

**Table 5:** Kit performance characteristics.

	Test Kit 1	Test Kit 2	Test Kit 3	Test Kit 4
Matrix–oats	Yes	No	No	Yes
Limit of detection	50 ppb	10 ppb	50 ppb	12 ppb
Quantification range	50–3000 ppb	10–800 ppb	50–500 ppb	10–360 ppb
Test time *	5 min	5 min	5 min	45 min
Ease of use	Easy	Easy	Easy	Technical skills required
Kit reagents supplied	All supplied	No extraction buffer	All supplied	All supplied
Cost per analysis	GBP 7.20	GBP 16.80	GBP 7.40	GBP 7.44
False negative rate (%)	1.1	7.6	2.2	6.5
False positive rate (%)	2.2	0	0	0
Recovery (ERM sample) (%)	93	79	102	69

* Incubation following sample preparation.

**Table 6:** Optimised MS/MS parameters for the analytes quantified.

Analyte	Precursor Ion (*m*/*z*)	Product Ion (*m*/*z*)	Declustering Potential (DV)	Collision Energy (eV)	Collision Cell Exit Potential
Aflatoxin B_1_	313.061 313.061	285.1 241.1	121 121	33 53	14 14
Aflatoxin B_2_	315.074 315.074	287.2 259.1	141 141	37 41	14 14
Aflatoxin G_1_	329.055 329.055	243.2 311.1	131 131	37 31	18 16
Aflatoxin G_2_	331.057 331.057	313 245.2	106 106	35 41	16 14
Deoxynivalenol	297.097 297.097	249.1 203.2	91 91	21 21	20 20
Fumonisin B_1_	722.316 722.316	704.3 334.4	1 1	41 53	38 10
Fumonisin B_2_	706.309 706.309	336.1 354.3	126 126	49 47	20 18
Ochratoxin A	404.092 404.092	239 358.1	81 81	33 21	12 18
T-2 Toxin	484.3 484.3	215.2 185.1	76 76	29 31	18 11
HT-2 Toxin	442.257 442.257	263.102 215.102	71 71	19 19	14 22
Zearalenone	317.1 317.1	175 131.1	−100 −100	−34 −42	−13 −8

**Table 7:** Chromatography gradient elution conditions.

Time (min)	MPA (%)	MPB (%)
0	99	1
1.0	99	1
3.0	50	50
9.0	1	99
11.5	1	99
12.0	99	1
14.0	99	1

**Table 8:** Validation spiking levels used.

Mycotoxin	HL Spike (μg/kg)	LL Spike (μg/kg)
Aflatoxin B_1_	5	1
Aflatoxin B_2_	5	1
Aflatoxin G_1_	5	1
Aflatoxin G_2_	5	1
Fumonisin B_1_	200	40
Fumonisin B_2_	200	40
Deoxynivalenol	200	40
Zearalenone	200	40
Ochratoxin A	10	2
T-2 toxin	200	40
HT-2 toxin	200	40

**Table 9:** Validation data.

Analyte	Mean Conc. (µg/kg)	StdDev	RSD (%)	RA (%)	RE (%)	LOD (ppb)	LOQ (ppb)
Aflatoxin B_1_	4.7	0.2	5.1%	93.7%	94.0%	0.1	0.5
Aflatoxin B_2_	5.1	0.3	6.0%	102.5%	96.8%	0.2	0.7
Aflatoxin G_1_	2.9	0.3	10.6%	57.9%	88.4%	0.1	0.4
Aflatoxin G_2_	3.3	0.3	9.2%	65.9%	91.3%	0.1	0.5
Deoxynivalenol	191.2	6.2	3.2%	95.6%	100.8%	3.7	12.4
Fumonisin B_1_	153.2	12.5	8.2%	76.6%	83.5%	4.2	13.8
Fumonisin B_2_	168.9	10.6	6.3%	84.5%	86.9%	2.6	8.7
Ochratoxin A	9.2	0.4	4.2%	91.9%	95.3%	0.3	1.0
T-2 Toxin	187.2	4.5	2.4%	93.6%	98.5%	3.4	11.4
HT-2 Toxin	188.1	4.8	2.6%	94.1%	97.7%	4.1	13.6
Zearalenone	186.1	6.3	3.4%	93.1%	99.5%	1.8	6.1

**Table 10:** Test Kit protocols.

	Test Kit 1	Test Kit 2	Test Kit 3	Test Kit 4
Sample size	10 ± 0.1 g	5 ± 0.1 g	10 g	5 g
Extraction	Add extraction powder. Add 50 mL distilled or deionized water. Shake vigorously for 3 min or blend for 1 min. Filter or centrifuge for 30 s at 2000× *g*.	Add 25 mL extraction buffer. Vortex for 2 min. Filter (≤5 min).	Add extraction powder. Add 37 mL distilled or deionized water. Shake for 1 min 30 s. Filter.	Add 25 mL of ready-to-use extraction buffer. Shake the sample for 10 min. Centrifuge for 10 min at 3000× *g*
Analysis	Mix 100 μL extract with 1500 μL sample diluent. Insert a test strip into the cartridge. Insert the cartridge into the reader. The barcode is read. Enter a sample ID. Add 400 μL sample extract.	Transfer 100 μL of filtered extract to the LFD sample well. Develop for 5 min. Insert the LFD into the reader.	Mix 600 μL of running buffer with 100 μL of sample extract. Transfer 150 μL to a tube and add the test strip. Develop for 5 min. Insert the test strip into the reader.	Dilute the supernatant 1:1 with methanol/distilled water (70/30; *v*/*v*). To 50 μL of standard or sample in separate duplicate wells, add 50 μL conjugate and 50 μL antibody. Mix and incubate at room temperature for 30 min. Wash 3 times with 250 μL wash buffer. Add 100 μL of substrate/chromogen to each well, mix and incubate for 15 min at room temperature in the dark. Add 100 μL of the stop solution to each well. Measure the absorbance at 450 nm.
Results	Results are displayed on the reader screen. Calibration cartridges are provided by the manufacturer.	Results are displayed on the reader screen. Automatic calibration through the barcode and lot ID of the strips.	Results are displayed on the reader screen. Automatic calibration through the barcode and lot ID of the strips.	Evaluation of results through software (absorbance values of the samples interpolated against those of the calibration curve). Calibration curve performed by the analyst using standards provided.

## Data Availability

The data presented in this study are available upon request from the corresponding author. The data are not publicly available to protect the privacy of the stakeholders and the kit manufacturers’ brands.
